# Metabolome Analysis under Aluminum Toxicity between Aluminum-Tolerant and -Sensitive Rice (*Oryza sativa* L.)

**DOI:** 10.3390/plants11131717

**Published:** 2022-06-28

**Authors:** Lihua Xie, Huijuan Li, Zhengzheng Zhong, Junjie Guo, Guocheng Hu, Yu Gao, Zhihua Tong, Meilan Liu, Songping Hu, Hanhua Tong, Peng Zhang

**Affiliations:** 1 State Key Laboratory of Rice Biology, China National Rice Research Institute, Hangzhou 310006, China; xielihua6658@163.com (L.X.); lihuijuan0812@163.com (H.L.); zhengzi711@163.com (Z.Z.); 15725929637@163.com (J.G.); cnrrihugc@163.com (G.H.); zkz0804@yeah.net (Y.G.); zhtong0628@163.com (Z.T.); zyzhang_311@163.com (M.L.); 2 Research Center of Plant Functional Genes and Tissue Culture Technology, College of Bioscience and Bioengineering, Jiangxi Agricultural University, Nanchang 330045, China

**Keywords:** rice (*Oryza sativa* L.), aluminum toxicity, metabolome analysis, lipids, phenolic acids, cysteine, putrescine

## Abstract

Aluminum (Al) solubilizes into trivalent ions (Al^3+^) on acidic soils, inhibiting root growth. Since about 13% of global rice cultivation is grown on acidic soils, improving Al tolerance in rice may significantly increase yields. In the present study, metabolome analysis under Al toxicity between the Al-tolerant variety Nipponbare and the Al-sensitive variety H570 were performed. There were 45 and 83 differential metabolites which were specifically detected in Nipponbare and H570 under Al toxicity, respectively. Furthermore, the results showed that 16 lipids out of 45 total metabolites were down-regulated, and 7 phenolic acids as well as 4 alkaloids of 45 metabolites were up-regulated in Nipponbare, while 12 amino acids and their derivatives were specifically detected in H570, of which 11 amino acids increased, including *L*-homoserine and *L*-methionine, which are involved in cysteine synthesis, *L*-ornithine and *L*-proline, which are associated with putrescine synthesis, and 1-aminocyclopropane-1-carboxylate, which is associated with ethylene synthesis. The contents of cysteine and s-(methyl) glutathione, which were reported to be related to Al detoxification in rice, decreased significantly. Meanwhile, putrescine was accumulated in H570, while there was no significant change in Nipponbare, so we speculated that it might be an intermediate product of Al detoxification in rice. The differential metabolites detected between Al-tolerant and -sensitive rice variants in the present study might play important roles in Al tolerance. These results provide new insights in the mechanisms of Al tolerance in rice.

## 1. Introduction

About 40% of the world’s total land area is acidic, and over 50% of arable land is distributed in acidic soil areas [1]. Moreover, about 13% of global rice cultivation is grown on acidic soils. Aluminum (Al) is the most abundant metal in the earth’s crust; it is dissolved from soil and released into the rice rhizosphere in the form of soluble Al^3+^ under pH values lower than 5.0 [2]. A high concentration of Al^3+^ can damage root systems, and inhibit the uptake of water as well as mineral nutrients, resulting in reduced rice yield [3,4,5,6]. Therefore, Al toxicity is considered a major limiting factor for rice production in acidic soils.

The response to Al toxicity in plants is an inducible process. There are two mechanisms of Al resistance in plants, i.e., internal and external Al detoxification [7,8]. In previous studies, it was found that organic ligands such as organic acids [9,10,11,12], proteins [13] and other phenols [14] could form stable complexes with Al^3+^ in the cell wall or membrane, so Al^3+^ could be transported into the vacuole, out of plasma membrane or fixed in cell walls for the completion of internal detoxification [7,8,15]. Furthermore, the release of phenolic compounds [14], the formation of an adhesive layer [16], and the exudation of organic acids [11], as well as the secretion of phosphate [17], can form a non-toxic complex with metal ions in the extracellular area, preventing Al from entering cells [15]. The Al-activated malate transporter (ALMT) releases malate anions [18,19,20,21], which, together with multidrug and toxic compound extrusion (MATE)-secreting organic acids [22,23], are the two major mechanisms against Al toxicity in external Al detoxification.

In rice (*Oryza sativa* L.), the genes associated with Al tolerance identified by molecular and genetic studies can be roughly grouped into the following categories [15]: the ALMT family (regulating malic acid exudation) [18,19,20,21], MATE family (mediating citric acid exudation) [22,23], ABC family membrane transporters (responsible for intracellular Al redistribution) [24,25,26,27], and the natural resistance-associated macrophage protein (Nramp family, involved in ion transport) [28,29]. Moreover, the transcription factors ART1 [30], ASR5 [31] and OsWRKY22 [32] were reported to be involved in regulating the mechanism of Al detoxification in rice [2,33,34]. Several genes related to Al tolerance in rice have been cloned, including *OsMGT1* (magnesium transporter) [35], *OsCDT3* (Al^3+^ binding protein) [30], *STAR1* and *STAR2* (bacterial ABC transporter) [25], *OsALS1* (ABC transporter) [24] and *OsEXPA10* (cell wall relaxation protein) [36], *OsFRDL2* (MATE family, compound excretion gene) [37], *OsFRDL4* (MATE family, citrate transporter) [38], and *Nrat1* (Nramp family, Al specific transporter gene) [28]. These studies suggest that Al tolerance in rice is regulated by multiple genes and is pretty complicated. Therefore, the mechanism of Al tolerance in rice still needs to be further studied and improved.

In recent years, omics techniques, e.g., genomics, proteomics, and metabolomics, have been applied to dissect the code of complex traits in plants. Based on qualitative and quantitative analysis of metabolites, metabolomics can be used to analyze metabolic pathways and networks, as well as the response mechanism of metabolites under stress. In the present study, we aimed to study the differences in metabolism level between Al-tolerant and -sensitive rice varieties through metabolome analysis and analyze the changes of metabolites before and after Al toxicity treatment, as well as supply some useful information for uncovering the mechanism of Al tolerance in rice.

## 2. Results

### 2.1. Identifying Al Tolerance of H570

**Figure 1:**
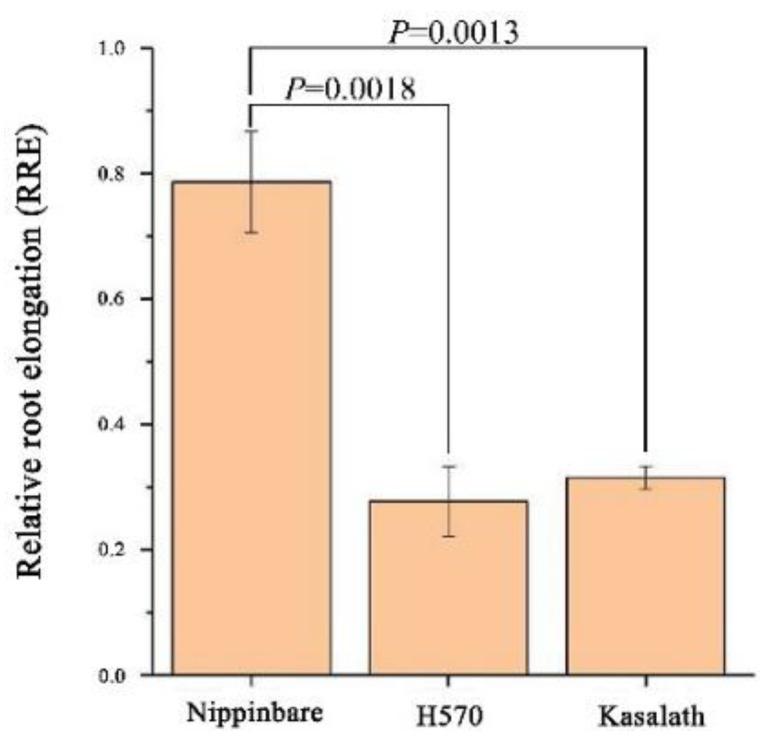
Relative root elongation (RRE) of three varieties in the present study.

The RREs (relative root elongation) of Nipponbare, H570, and Kasalath under Al toxicity were evaluated. The Al tolerance of Nipponbare and Kasalath was identical to that in previous studies, and the RRE of H570 was 0.27, which showed H570 to be Al-sensitive (Figure 1).

### 2.2. Qualitative and Quantitative Analysis of Metabolites

In the present study, log_2_ FC (fold change) was used to demonstrate regulatory levels of metabolites. The positive value of log_2_ FC showed that the metabolites were up-regulated, while the negative value showed that they were down-regulated.

The values of log_2_ FC and VIP (variable importance in projection) values of the OPLS-DA (orthogonal partial least squares discriminant analysis) models were combined to screen differential metabolites. The above 611 metabolites were screened according to the following criteria: (1) metabolites with absolute log_2_ FC ≥ 1 were selected. (2) based on the above, metabolites with VIP ≥ 1 were selected.

**Figure 2:**
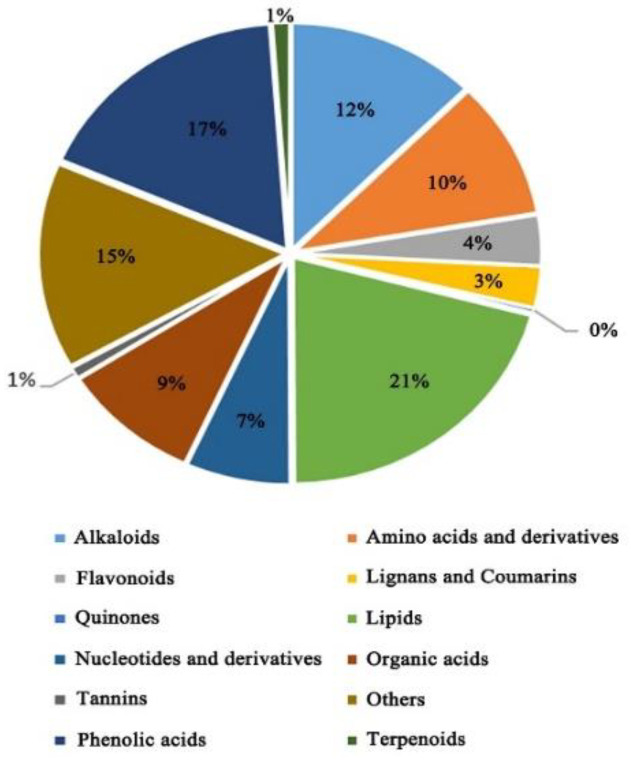
Metabolites’ classification.

A total of 611 metabolites were detected based on a UPLC-MS/MS (high-performance liquid chromatography–tandem mass spectrometry) detection platform and self-built database (Table S1). According to the classification of the first group of substances, these metabolites can be divided into nine groups (Figure 2).

### 2.3. Multivariate Analysis of Metabolites

**Figure 3:**
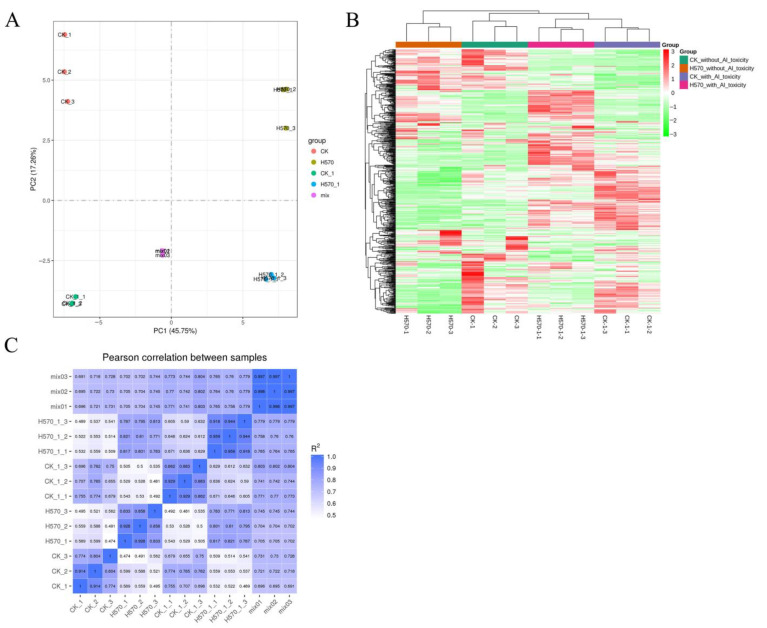
PCA, cluster analysis, and correlation coefficients among three biological replications. (**A**) PCA. PC1 represents the first principal component, PC2 represents the second principal component; (**B**) Cluster analysis. The horizontals are sample names, the verticals are the metabolite information, different colors are the values obtained after the standardization of relative content (red represents high content, green represents low content); (**C**) The correlation coefficients. CK represents Nipponbare without Al toxicity, H570 represents H570 without Al toxicity, CK-1 represents Nipponbare with Al toxicity, H570-1 represents H570 with Al toxicity, and mix represents quality control samples. The sample numbers in all of the following figures are the same.

Principal component analysis (PCA) was performed on all samples (including QC (quality control) samples) to determine the separation trend of metabolites between groups and whether there were differences in metabolites within groups. The analysis showed little difference in the inter-group metabolome between the different varieties, while there was a clear separation trend between Nipponbare and H570 (Figure 3A). At the same time, we also carried out a cluster hierarchical analysis, which showed, intuitively, the differential metabolome between the two varieties (Figure 3B). The PCA and cluster analysis showed that the two varieties had different metabolic profiles and indicated high repeatability within all samples. Moreover, we found that the *R^2^* (PCC (Pearson correlation coefficients)) ranged from 0.774 to 0.959, which indicated a high correlation among the three biological replications (Figure 3C).

### 2.4. Analysis of Differential Metabolites with and without Al Toxicity Treatment

**Figure 4:**
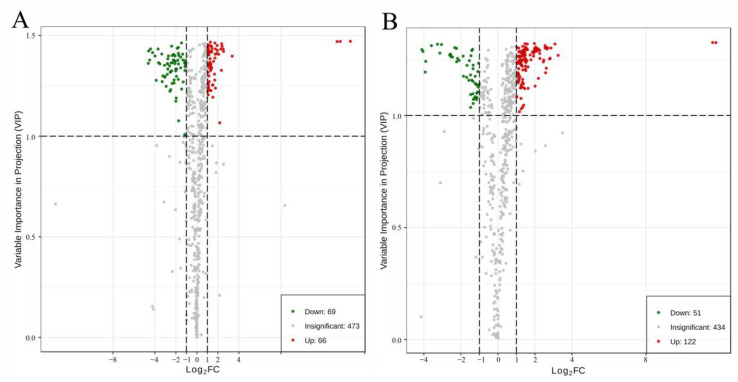
Volcano plot of differential metabolites. Each point in the volcano plot represents a metabolite, and the horizontal coordinate represents the log value of the quantitative difference multiple of a certain metabolite in two samples. The vertical axis represents the VIP value. (**A**) Volcano plot of differential metabolites in Nipponbare; (**B**) Volcano plot of differential metabolites in H570.

**Figure 5:**
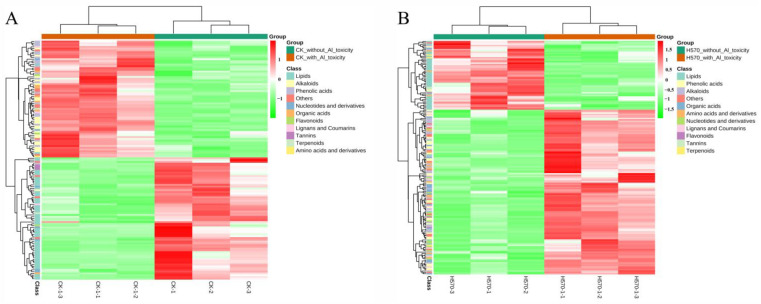
Cluster heat map of differential metabolites. The horizontal is the sample name, the vertical is the information of differential metabolites, the clustering tree on the left of the figure is the clustering tree of differential metabolites, and different colors are the values obtained after standardized treatment of relative content (red represents high content, green represents low content), Group is the experimental group, and Class is the categories of different substances. (**A**) Cluster heat map of differential metabolites in Nipponbare; (**B**) Cluster heat map of differential metabolites in H570.

There were 135 differential metabolites screened from Nipponbare, among which 69 metabolites decreased and 66 metabolites increased under Al toxicity (Figure 4A, Table S2). Additionally, 173 differential metabolites were detected from H570, among which 51 decreased and 122 increased under Al toxicity (Figure 4B, Table S3). The inter-group distribution of these differential metabolites is shown as Z-score plots (Figures S1 and S2). To detect the rule of changing metabolites under Al toxicity, a cluster hierarchical analysis was conducted on metabolites with significantly increased or decreased levels. The profile of metabolism between Nipponbare and H570 before Al treatment was not quite common, i.e., the contents of many metabolites, e.g., phenolic acids, alkaloids, organic acids, amino acids and their derivatives, flavonoids, nucleotides and their derivatives, lignans and coumarins, tannins and quinones, were different (Figure S3A). However, it was interestingly found that there was no significant change in the content of lipids in the two varieties before Al toxicity treatment (Figure S3A), but the contents of lipids in H570 were obviously higher than that of Nipponbare after Al toxicity treatment (Figure S3B). Furthermore, it could be summarized that the metabolism level between Nipponbare and H570 after Al treatment versus without Al treatment were quite differential (Figure 5). As can be seen in Figure 5, increased metabolites in H570 were more than those in Nipponbare. After nine days under Al toxicity, the contents of most phenolic acids, alkaloids, organic acids, and other metabolites increased in H570 and Nipponbare, while the contents of lipids decreased in both varieties (Figure 5).

According to log2 FC and VIP values of the differential metabolites, seven metabolites ranked in the top 20 in Nipponbare were found, including three phenolic acids, two alkaloids, one lipid and one other (Table 1). In H570, 11 metabolites with two values ranked in the top 20 were found, including three lipids, two alkaloids, one amino acid and derivatives, one phenolic acid, one flavonoid and three others; the corresponding information is shown in Table 2.

### 2.5. Venn Analysis of Differential Metabolites

**Figure 6:**
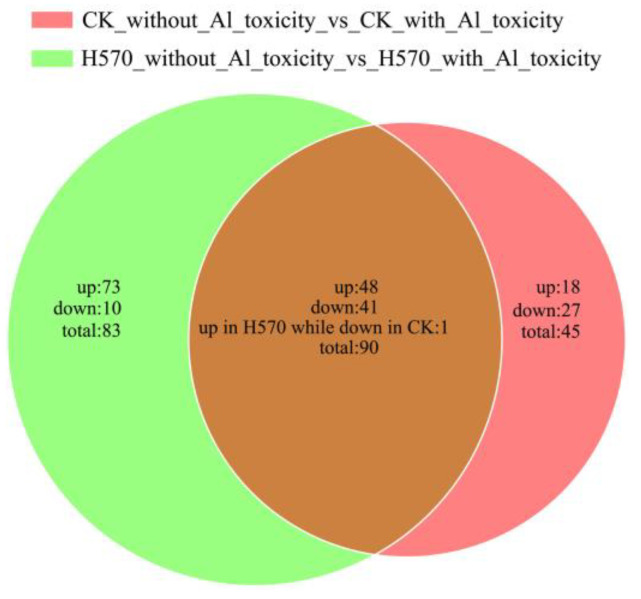
Venn diagram showing the numbers of differential metabolites between Nipponbare and H570. CK represents Nipponbare. Each circle in the figure represents a comparison group, the number in the overlapped parts represents the number of common differential metabolites between the comparison groups, and the number in the non-overlapped parts represents the number of unique differential metabolites in the comparison group.

The relationship between different metabolites in Al-tolerant and -sensitive varieties is demonstrated in the form of a Venn diagram. There were 90 common differential metabolites between Nipponbare and H570. Except for one metabolite i.e., pmn001378 (terpineol mono-glucoside is belonging to lignans) had different regulatory levels; the remaining 89 differential metabolites maintained the identical regulatory levels. More than half (48/89, most of which were phenolic acids, alkaloids and organic acids) were up-regulated, while most of the lipids (35/37) and all of the tannins (3) were down-regulated in two varieties (Figure 6 and Table S4).

Besides, more than half (27/45, most of which were lipids) of Nipponbare-specific metabolites were down-regulated, while most H570-specific metabolites were up-regulated after Al toxicity treatment. Moreover, 18 Nipponbare-specific metabolites were up-regulated (most of which were phenolic acids and alkaloids) under Al toxicity. After Al toxicity, phenolic acids, amino acids, nucleotides and their derivatives, organic acids, alkaloids and some free fatty acids were accumulated in Al-sensitive variety, H570 (Figure 6 and Table S4). However, cysteine, alanylleucine and s-(methyl) glutathione, which have been reported to be related to Al detoxification in rice [39,40,41], were significantly down-regulated.

### 2.6. K-Means Clustering Analysis of Differential Metabolites

**Figure 7:**
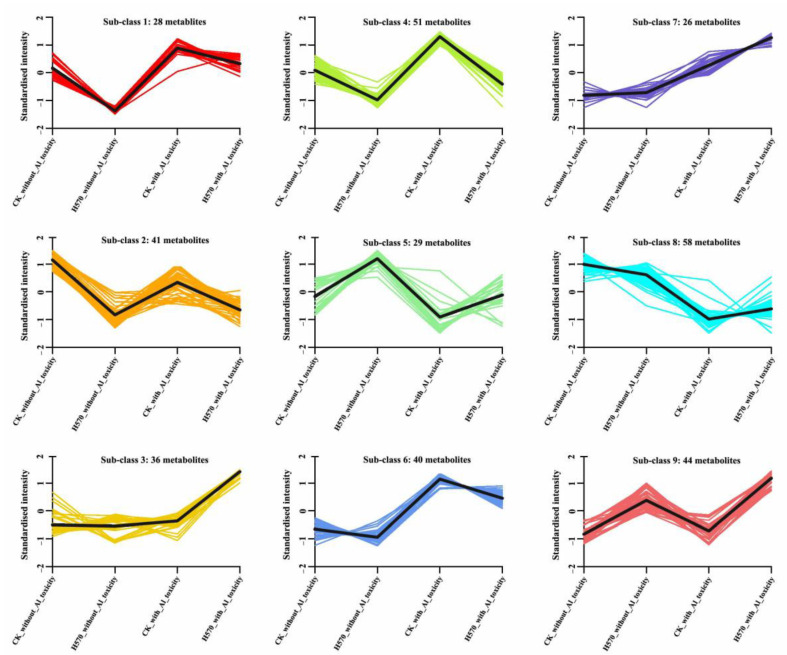
K-means map of differential metabolites in Nipponbare and H570. The abscissa represents sample name, the ordinate represents the relative content of standardized metabolites.

We conducted a k-means clustering analysis to study the changing trend of the relative content of metabolites in Nipponbare and H570. A total of nine clusters were obtained. It can be seen in Figure 7 that the changing trend of the corresponding differential metabolites in the Sub-classes 1, 2, 4, 5, 6 and 9 before and after Al toxicity were common between Nipponbare and H570. Besides, the relative contents of the differential metabolites in the Sub-classes 1, 4 and 6 increased under Al toxicity, while the metabolites in Sub-class 5 decreased. The 41 metabolites in Sub-class 2 clusters were down-regulated in Nipponbare under Al toxicity, but significant changes of these metabolites were not detected in H570. Furthermore, it was discovered that most of the above 41 metabolites were alkaloids, organic acids, and phenolic acids. In Sub-class 9 clusters, there was no significant change in the relative contents of 44 metabolites before and after Al toxicity in Nipponbare, while the expression of 44 metabolites in Sub-class 9 was up-regulated under Al toxicity in H570, and most of them were phenolic acids and flavonoids (Figure 7 and Table S5).

The changing trend of the metabolites in the remaining three clusters differed greatly between Nipponbare and H570 under Al toxicity. Most of the relative contents of the 35 metabolites in Sub-class 3 were common between Nipponbare and H570 before Al toxicity. However, the contents in Nipponbare increased a little under Al toxicity, while the contents in H570 increased greatly. Phenolic acids accounted for a large proportion of these up-regulated metabolites. The relative contents of the 26 differential metabolites in Sub-class 7 showed no significant change between Nipponbare and H570 before Al toxicity, but they showed an obvious rise after Al toxicity, especially for H570. Although the relative content of 58 metabolites in Sub-class 8 decreased in both varieties under Al toxicity, the trend of change was not consistent. The relative content of H570 was lower than that of Nipponbare before Al toxicity but higher than that of Nipponbare after Al toxicity. Meanwhile, it should be noted that nearly 83% of these metabolites were lipids; lysophosphatidylcholine (LPC, 20/48), lysophosphatidylethanolamine (LPE, 16/48), glyceride (10/48) and free fatty acid (2/48) were included (Figure 7 and Table S5).

### 2.7. KEGG Functional Annotation and Enrichment Analysis of Differential Metabolites

**Figure 8:**
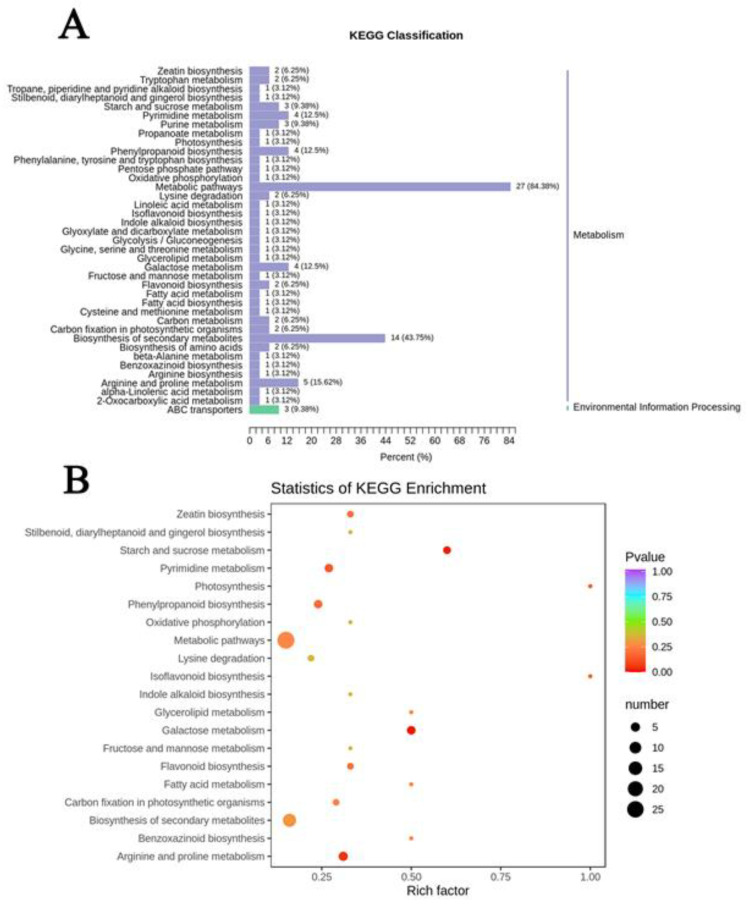
Enrichment analysis of differential metabolites on KEGG metabolic pathway in Nipponbare. (**A**) KEGG classification map of differential metabolites. The ordinate is the name of KEGG metabolic pathway, and the abscissa is the number of metabolites annotated to one pathway and their proportion to the total number of metabolites annotated; (**B**) KEGG enrichment map of differential metabolites. The abscissa represents the Rich factor corresponding to each pathway, the ordinate represents the pathway name, and the point with different color is *p*-value.

**Figure 9:**
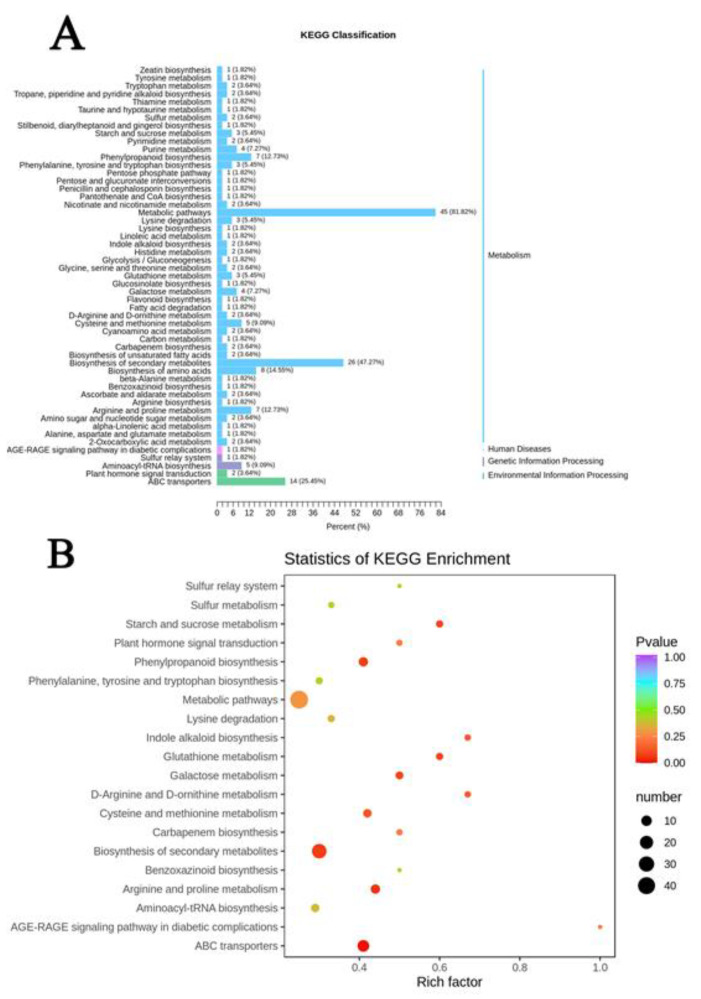
Enrichment analysis of differential metabolites on KEGG metabolic pathway in H570. (**A**) KEGG classification map of differential metabolites; (**B**) KEGG enrichment map of differential metabolites.

In the contrast experimental group of Nipponbare, there were 32 metabolites annotated by KEGG (Kyoto encyclopedia of genes and genomes) with significant changes, distributed into 39 pathways (Figure 8A). Of these metabolites, 83% were involved in metabolic pathways and 44% in galactose metabolism (Ko00052, *p* = 0.014); starch and sucrose metabolism (Ko00500, *p* = 0.019) were significantly enriched compared with other pathways (Figure 8B). The former is involved in the regulation of galactose, saccharose, sucrose and fructose 6-phosphate, while the latter regulates fructose 6-phosphate, sucrose and trehalose. Moreover, the contents of polysaccharides except for fructose 6-phosphate were up-regulated under Al toxicity. There were 55 differential metabolites annotated by KEGG and 54 pathways were associated with H570 (Figure 9A). The differential metabolites accounted for 81.82 and 47.27%, and were annotated in metabolic pathways and the biosynthesis of secondary metabolites, respectively. In addition, 14 differential metabolites, annotated as ABC transporters, accounted for 25.45% in this group, and this pathway was the most significantly enriched with a value of *p* = 0.0107 (Figure 9B). Information related to all of the above metabolic pathways is shown in Tables S6–S9.

**Figure 10:**
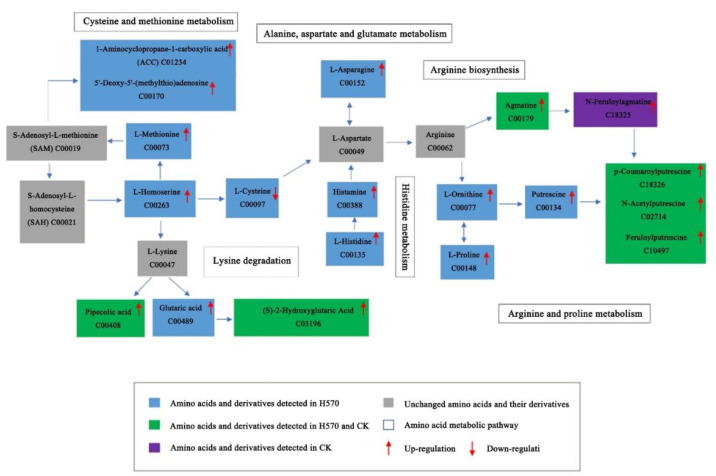
Metabolic pathways of amino acids and their derivatives.

Under Al toxicity stress, amino acids and their derivatives were one of the most differential metabolites between Nipponbare and H570. *L*-cysteine, s-(methyl) glutathione, alanyl-leucine, other amino acids and their derivatives (*L*-homoserine, *L*-methionine, *L*-histidine, *L*-ornithine, *L*-proline, histamine, *L*-asparagine, pipecolic acid, N-acetylglycine, N-acetyl-*L*-leucine, 3-hydroxy-3-methylpentane-1,5-dioic acid) were up-regulated under Al toxicity in H570, while only pipecolic acid was screened in Nipponbare with the same regulation level. The metabolic pathways associated with above metabolites are shown in Figure 10.

## 3. Discussion

Nipponbare, a widely recognized variety with strong Al tolerance, was used as a positive control, and Kasalath, a reported variety with sensitivity to Al toxicity, was used as a negative control to study the RRE after treatment with 150 μM Al toxicity (pH = 4.0) for 1 day. The RRE values of Nipponbare, H570 and Kasalath were 0.78, 0.27 and 0.32, respectively, which indicates that H570 was sensitive to Al toxicity.

Al^3+^ with a high concentration could damage root systems, and inhibit the uptake of water as well as mineral nutrients. Al tolerance in plants is a complex process including changes of “Biological process”, “Cellular component” and “Molecular function”, which might be mediated by various factors including metabolites. Moreover, many metabolites in other plants have been proven that they are helpful to detoxify the threat from Al. Thus, we speculated that rice could alleviate the harm from Al by modulating its metabolome under Al toxicity. In the present study, the Al-sensitive variety H570 and Al-tolerant variety Nipponbare were used as materials to identify their differential metabolites. A total of 135 and 173 differential metabolites were screened by metabonomics analysis of Nipponbare and H570, respectively. Among these metabolites, 89 metabolites were detected in both varieties with the same regulatory level. We speculated that these metabolites might be the basic metabolites for rice growth and not be affected by Al toxicity. There were 45 and 83 differential metabolites specifically screened in Nipponbare and H570, respectively; these metabolites and their metabolic pathways might be involved in the regulation of Al-tolerant phenotypes. We also found that 73 out of 83 different metabolites in H570 were up-regulated under Al toxicity. This phenomenon of the high accumulation and low decomposition of metabolites in root tip cells might be one of the reasons why H570 showed Al-sensitivity when it was exposed to Al toxicity.

Al tolerance of rice is achieved through the elimination of Al from the root tip, and polysaccharides in the cell wall might play an important role in the specific elimination of Al from the root tip of rice [42]. Above study proved that the lower the content of polysaccharides in the cell wall is, the higher the degree of methylation would be, and the lower the content of carboxyl is, the lower the binding degree of Al to the cell wall would be. In the present study, it was found that some sugars and sugar alcohols in Nipponbare and H570 were up-regulated under Al toxicity, but the change in H570 was significantly higher than that in Nipponbare. Meanwhile, Al can increase the permeability of the plasma membrane when combined with the negative potential of the plasma membrane surface of rice root cells [43]. The negative charge on the plasma membrane surface is mainly caused by phospholipids. Therefore, the ratio of phospholipids in the plasma membrane is closely related to the Al tolerance of rice. Reducing the ratio of phospholipids in the substrate can improve the Al tolerance of rice. In the present study, lipid substances in both Al-tolerant and Al-sensitive varieties were down-regulated, but the relative lipid content of H570 under Al toxicity was higher than that of Nipponbare, which might be a factor for the inhibition of H570 by Al toxicity.

Previous studies have suggested that cysteine is a key substrate for glutathione (GSH) biosynthesis, and it is a major factor limiting the production of GSH in plants [39]. Glutathione s-transferase (GST) catalyzes the transfer of GSH to a co-substrate containing a reactive electrophilic center to form a polar s-glutathione reaction product [40]. It helps cells in removing Al-induced reactive oxygen species (ROS) which had been proved to be related to Al tolerance [41] and toxins [44]. Cysteine is also a precursor to s-adenosylmethionine (SAM) biosynthesis, which is a subsequent substrate to 1-aminocyclopropane-1-carboxylate (ACC) biosynthesis, which is then converted to ethylene (ethylene associating with Al tolerance in rice was reported in a previous study [41] by the means of the ACC oxidase, thereby inhibiting tap root elongation [40]). In H570, the relative contents of cysteine and s-(methyl) glutathione decreased after Al toxicity treatment, while the content of ACC increased, which might be related to the reduction of RREs after Al toxicity treatment.

Yu et al. [45,46,47] showed that putrescine could alleviate Al-induced oxidative stress in wheat roots by reducing Al-induced hydrogen peroxide accumulation in wheat root tips and by inhibiting NADPH oxidase in the plasma membrane of Al-stressed wheat. The Al inhibition on wheat seedling roots was alleviated by the addition of putrescine through decreasing the content of polysaccharides in the cell wall and increasing the methylation degree of pectin in the cell wall [42]; thus, putrescine could reduce the accumulation of Al in the cell wall of root tip in wheat. In the present study, we found that there was no significant change of putrescine in Nipponbare before and after Al toxicity treatment. However, the relative content of putrescine was up-regulated under Al toxicity in H570, which was different from the results of Yu et al. Therefore, we hypothesized that putrescine might be an intermediate product that enhances Al resistance in rice, and that its subsequent metabolic pathway is normal in Nipponbare, but inhibited in H570.

In addition to alkaloids, amino acids and their derivatives, phenolic acids, and saccharides as well as alcohols, some novel metabolites contributing to Al tolerance in rice might be dissected in many classes of metabolites such as free fatty acids, lignans, nucleotides, as well as organic acids (Table S4), which showed many different trends between Nipponbare and H570, and these were not reported in previous studies. There are many groups with negative charges in lignans, which have a strong affinity to metal ions in the soil. In the present study, the contents of five lignans in H570 were specifically detected to increase under Al toxicity. Interestingly, terpineol mono-glucoside (pmn001378), which is supposed to be one of the lignans, was up-regulated in H570, but it was down-regulated in Nipponbare. We speculated that the above five lignans which might carry negative charges had a strong affinity to Al^3+^, which increased the amount of Al^3+^ absorbed into cells through the cell wall and inhibited the root growth of H570.

This study revealed the difference in metabolic regulation between H570 and Nipponbare under Al toxicity and analyzed the process of the Al-induced metabolome change of the Al-sensitive variety H570. The large accumulation of phenolic acids, alkaloids, organic acids and flavones in H570 might be one of the factors that make H570 greatly Al-sensitive under Al toxicity. In addition, the contents of some lipids and sugars which have been reported to be related to rice Al tolerance in our transcripomics study [48] in H570 were higher than those in Nipponbare, which might increase the binding ability of the cell wall to Al^3+^ and affect the scavenging ability of the cell wall to Al^3+^. The decrease in cysteine content in H570 repressed the synthesis of GSH, and then reduced S-(methyl) glutathione content; an increased ACC content might improve the ethylene content of H570 and inhibit taproot elongation. Interestingly, the content of putrescine increased significantly in H570, which might influence its alleviating effect on oxidative stress and its promotion of the metabolism of cell wall polysaccharides and the degree of pectin methylation.

## 4. Conclusions

Through the metabolomics analysis, it is speculated that the lower polysaccharide and lysophospholipids material can improve the resistance of rice to Al. The metabolism promotes the transformation of *L*-serine to cysteine, methionine to s-(methyl)glutathione, the decomposition of *L*-ornithine and *L*-proline, and the decomposition of putrescine in glutathione metabolism, which might also improve the Al tolerance of rice. Moreover, through down-regulating the lipids and up-regulating the phenolic acids as well as alkaloids, which were specifically detected in Nipponbare, this might improve the Al tolerance of rice.

## 5. Materials and Methods

### 5.1. Plant Material

Relative root elongation, RRE was used to evaluate the Al tolerance of one rice variety; please see more details in our previous study [49]. It is referenced that one rice variety is supposed to be Al-sensitive if the RRE is less than 0.50 in the seedling stage [50].

Nipponbare, which is commonly considered as one Al-tolerant variety, and one Al-sensitive variety H570, which is one of the parents of the super hybrid rice Zhongzheyou 1, were chosen for metabolome analysis. Besides, Kasalath, which has been proven to be Al-sensitive (RRE = 0.32) [25,30,51], was selected as a negative control for identifying Al-sensitive varieties.

### 5.2. Sample Preparation and Extraction

In the present study, the seeds firstly were sterilized with 1% H_2_ O_2_ solution for 30 min, and then washed with deionized water three times. We spread the sterilized seeds on Petri dishes, then added deionized water to soak them overnight in a 30 °C incubator (full intelligent artificial climate plant box, HP1500 GS-B). We then transferred the Petri dishes to a dark incubator for seeds, germinating them for two days, and selected uniform seeds and transferred them into a 96-well plate in a 1 L plastic container with the conditions of 14 h light (30 °C, 70.5%RH, 20,000 Lx)/10 h darkness (28 °C, 70.5%RH, 0 Lx). Rice seedlings were cultured with 0.5 mM CaCl_2_ solution (pH 4.0) as a control, while seedlings were treated with 0.5 mM CaCl_2_ and 150 μM AlCl_3_ solution (pH 4.0). Three replications were performed and the root length of 20 seedlings in each treatment was measured with a ruler before and after treatments (24 h). Roots were collected and immediately freeze-dried with liquid nitrogen on the 9th day of culture. The freeze-dried root of rice was treated, extracted and filtered according to the method described in the study of Zou et al. [52], before UPLC-MS/MS (high performance liquid chromatography-tandem mass spectrometry) analysis.

### 5.3. UPLC-ESI-Q TRAP-MS/MS Analysis

The samples were analyzed by a UPLC-ESI-MS/MS system (UPLC, Shim-pack UFLC SHIMADZU CBM30 A system, www.shimadzu.com.cn/, accessed on 20 May 2020; MS, Applied Biosystems 4500 Q TRAP, www.appliedbiosystems.com.cn/, accessed on 20 May 2020). The analytical conditions and sample measurements gradient program were followed as described by Zou et al. [52]. The UPLC effluent was alternatively connected to an ESI-triple quadrupole-linear ion trap (QTRAP)-MS (equipped with an ESI Turbo Ion-Spray interface, operating in positive and negative ion mode and controlled by Analyst 1.6.3 software). The ESI source operation parameters were referred to in the study of Chen et al. [53]. The MWDB (Metware database, http://www.metware.cn/) and MRM (multiple reaction monitoring) were used for qualitative and quantitative analysis of metabolites, respectively, following their standard metabolic operating procedures [54].

### 5.4. Statical Analysis

PCA and supervised multivariate OPLS-DA were performed on metabolite data using statistical functions in *R* software [55]. VIP values were extracted from the OPLS-DA result and were generated using the *R* package MetaboAnalyst [56]. The significantly regulated metabolites between groups were determined by VIP ≥1 and absolute log_2_ FC (fold change) ≥1 [57].

Heat maps were drawn by PheatMap software in the *R* package, and HCA (hierarchical cluster analysis) was conducted on the accumulation patterns of metabolites among different samples. The PCCs (Pearson correlation coefficients) between samples were calculated by the cor function in the *R* package and presented by the heatmap drawn by PheatMap software of the *R* package.

The metabolites detected were labeled through the KEGG (Kyoto encyclopedia of genes and genomes) compound database (http://www.kegg.jp/kegg/compound/, accessed on 25 May 2020), and the annotated metabolites were mapped into the KEGG pathway database (http://www.kegg.jp/kegg/pathway.html, accessed on 5 June 2020). Additionally, pathways that significantly regulated metabolites were fed into MSEA (metabolite sets enrichment analysis), and their significance was determined by *p*-values of hypergeometric tests.

## Figures and Tables

**Table 1:** The differential metabolites of Nipponbare with |Log_2_ FC| values and VIP values ranked in the top 20.

Index	Compounds	Class I	Class II	Peak Area			Peak Area			VIP Value	Fold_Change	Log2 FC	*p* Value *t-*Test	Type
				CK-1	CK-2	CK-3	CK-1-1	CK-1-2	CK-1-3					
Zmhn002334	6-O-Feruloyl-glucose *	phenolic acids	phenolic acids	9	9	9	227,960	221,680	228,460	1.47	25,114.81481	14.62	4.65 × 10^−5^	up
pmn001672	Furanofructosyl-α-D-(3-mustard acyl)glucoside	phenolic acids	phenolic acids	9	9	9	98,538	123,170	127,000	1.47	12,915.11111	13.66	2.92 × 10^−3^	up
pmn001671	Furanofructosyl-α-D-(6-mustard acyl)glucoside	phenolic acids	phenolic acids	9	9	9	145,410	72,407	65,020	1.47	10,475.44444	13.35	3.33 × 10^−2^	up
mws1080	Galactinol *	others	saccharides and alcohols	88,625	90,779	68,725	442,690	367,210	463,140	1.46	5.130557089	2.36	5.06 × 10^−3^	up
pmb1096	Indole	alkaloids	plumerane	409,440	386,920	498,240	1,951,300	1,486,000	1,498,600	1.45	3.812683454	1.93	9.15 × 10^−3^	up
pmb0490	p-Coum–aroylputrescine	alkaloids	phenolamine	591,020	477,430	636,660	3526,500	2,265,100	3,138,100	1.44	5.237022831	2.39	9.38 × 10^−3^	up
Lmhp009890	LysoPC 20:3	lipids	LPC	6,123,600	2,431,100	2,568,200	145,090	131,420	243,260	1.43	0.04672972	−4.42	5.08 × 10^−2^	down

Note: “*” represents that this compound is a isomer which can not be distinguished through mass spectrometry.

**Table 2:** The differential metabolites of H570 with |Log_2_ FC| values and VIP values ranked in the top 20.

Index	Compounds	Class I	Class II	PEAK Area			Peak Area			VIP Value	Fold_Change	Log2 FC	*p* Value *t-*Test	Type
				H570-1	H570-2	H570-3	H570-1-1	H570-1-2	H570-1-3					
pmb0500	N-p-Coumaroyl-N’-feruloylputrescine	alkaloids	phenolamine	9	9	9	32,722	20,015	30,617	1.33	3087.185185	11.59	9.73 × 10^−3^	up
XMP3554	HMBOA	alkaloids	alkaloids	9	9	9	53,112	22,603	18,683	1.33	3496.222222	11.77	5.09 × 10^−2^	up
mws1080	Galactinol *	others	saccharides and alcohols	165,860	145,240	128,560	1,472,600	1,185,600	1,061,500	1.32	8.460401219	3.08	5.08 × 10^−3^	up
pme0195	*L*-Cysteine	amino acids and derivatives	amino acids and derivatives	1,709,400	1,631,000	1,297,400	115,090	200,150	167,530	1.32	0.104094614	−3.26	4.75 × 10^−3^	down
Lmhp009802	LysoPE 20:3(2 n isomer) *	lipids	LPE	408,080	371,710	367,370	54,441	45,010	39,522	1.32	0.121145263	−3.04	3.42 × 10^−4^	down
Lmhp009890	LysoPC 20:3	lipids	LPC	2,757,700	3,972,800	2,445,600	314,980	236,130	210,140	1.31	0.082960081	−3.59	1.34 × 10^−2^	down
pme0519	D-(+)-Sucrose *	others	saccharides and alcohols	169,900	183,510	139,150	1,163,900	866,320	843,040	1.31	5.833319799	2.54	7.82 × 10^−3^	up
ML10181668	CYCLOLEUCINE	others	others	58,114	68,032	75,667	576,960	336,390	317,700	1.30	6.099953918	2.61	3.01 × 10^−2^	up
pmd0146	LysoPC 20:2(2 n isomer) *	lipids	LPC	483,220	774,760	456,840	93,220	124,720	123,530	1.30	0.199128772	−2.33	2.12 × 10^−2^	down
Hmbn005951	1,3-O-Di-p-Coumaroylglycerol	phenolic acids	phenolic acids	48,318	30,843	36,720	315,380	254,790	179,330	1.30	6.46784201	2.69	1.42 × 10^−2^	up
pmp000804	Isobavachalcone D	flavonoids	chalcones	37,408	22,536	22,904	223,310	221,930	157,150	1.30	7.271026458	2.86	6.44 × 10^−3^	up

Note: “*” represents that this compound is a isomer which can not be distinguished through mass spectrometry.

## Data Availability

The datasets used during the current study are available from the corresponding author on reasonable request.

## Supplementary Materials

The following supporting information can be downloaded at: https://www.mdpi.com/article/10.3390/plants11131717/s1, Figure S1: Z-score plot of differential metabolites in Al-tolerant varieties Nipponbare; Figure S2: Z-score plot of differential metabolites in Al-sensitive varieties H570; Figure S3: Cluster heat map of differential metabolites. Horizontal is the sample name, vertical is the information of differential metabolites, the clustering tree on the left of the figure is the clustering tree of differential metabolites, different colors are the values obtained after standardized treatment of relative content (red represents high content, green represents low content), Group is the experimental group, and Class is different substance categories. A. Cluster heat map of differential metabolites between Nipponbare and H570 before Al treatment; B. Cluster heat map of differential metabolites between Nipponbare and H570 after Al treatment. Table S1: The list of 611 metabolites detected in the present study; Table S2: Differential accumulation of metabolites between Nipponbare without Al toxicity and Nipponbare with Al toxicity; Table S3: Differential accumulation of metabolites between H570 without Al toxicity and H570 with Al toxicity; Table S4: Venn diagram distribution table of differential metabolites of Al-tolerant and -sensitive varieties before and after Al treatment; Table S5: K-means clustering information of differential metabolites in the present study; Table S6: KEGG enrichment statistics of Al-tolerant varieties Nipponbare; Table S7: Statistical analysis of KEGG differential enrichment classification of Al-tolerant varieties Nipponbare; Table S8: KEGG enrichment statistics of Al-sensitive varieties H570; Table S9: Statistical analysis of KEGG differential enrichment classification of Al-sensitive varieties H570.
