# Supplementary material for: Metabolome Analysis under Aluminum Toxicity between Aluminum-Tolerant and -Sensitive Rice (Oryza sativa L.)
Source: Plants (Basel). 2022 Jun 28;11(13):1717. doi: 10.3390/plants11131717 (PMC9269133; doi:10.3390/plants11131717)
Supplement: Supplementary file 1 [file plants-11-01717-s001.zip › plants-1738266_supplementary-done.pdf]

plants

MDPI

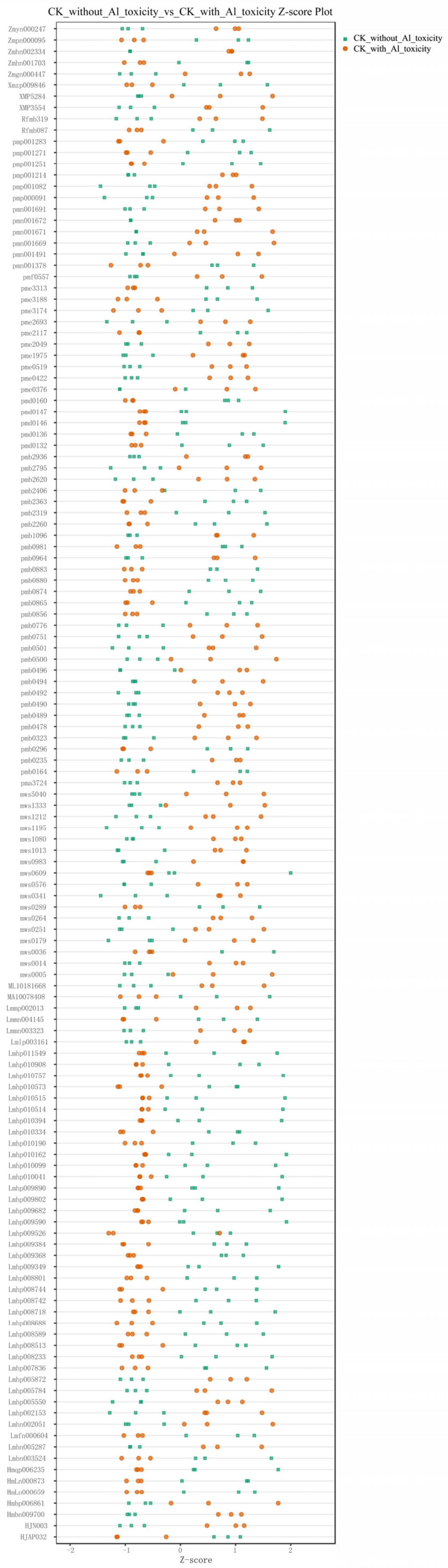
CK_without_Al_toxicity_vs_CK_with_Al_toxicity Z-score Plot
CK_without_Al_toxicity
CK_with_Al_toxicity
Z-score

Figure S1. Z-score plot of differential metabolites in Al-tolerant varieties Nipponbare.

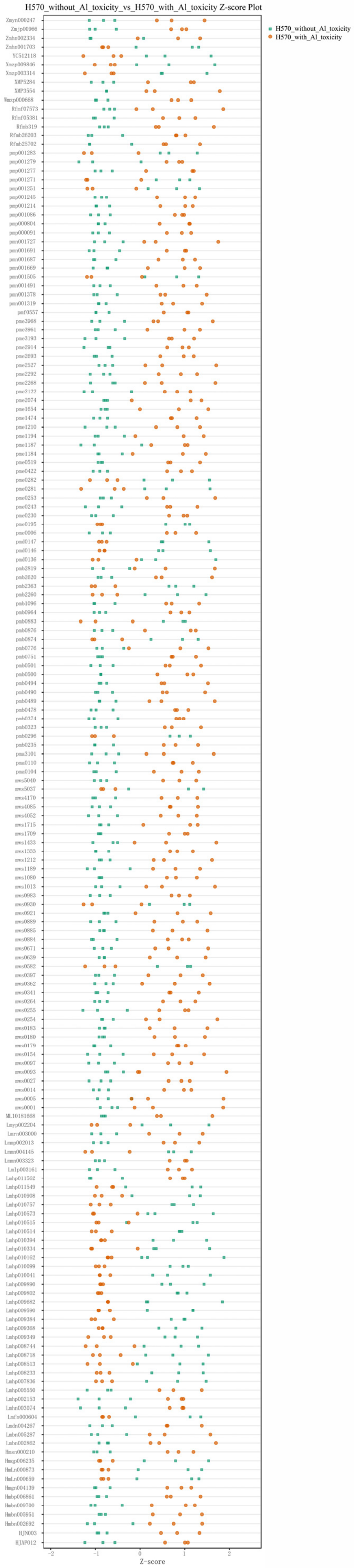
H570_without_Al_toxicity_vs_H570_with_Al_toxicity Z-score Plot
H570_without_Al_toxicity
H570_with_Al_toxicity
Z-score

Figure S2. Z-score plot of differential metabolites in Al-sensitive varieties H570.

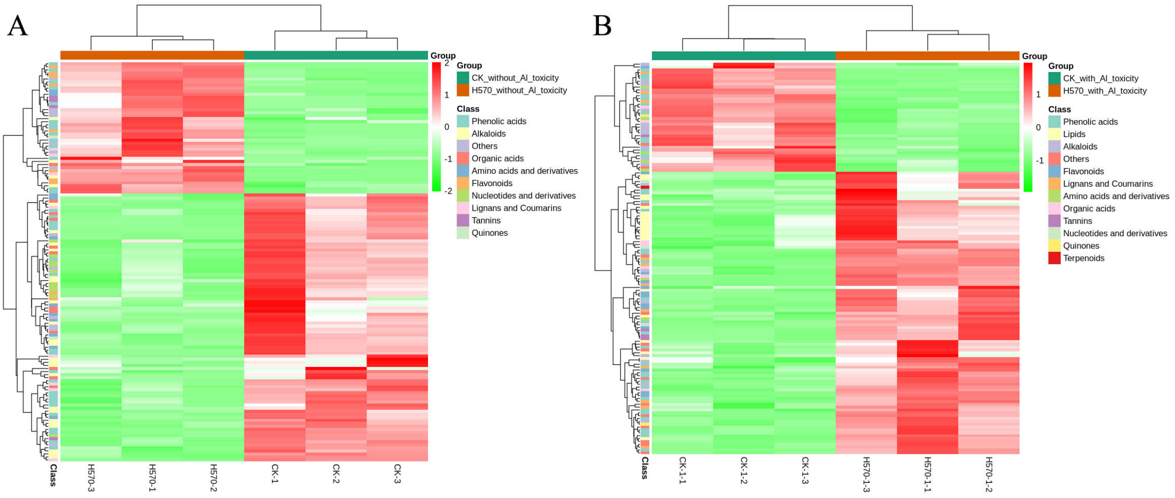

Figure S3. Cluster heat map of differential metabolites. Horizontal is the sample name, vertical is the information of differential metabolites, the clustering tree on the left of the figure is the clustering tree of differential metabolites, different colors are the values obtained after standardized treatment of relative content (red represents high content, green represents low content), Group is the experimental group, and Class is different substance categories. A. Cluster heat map of differential metabolites between Nipponbare and H570 before Al treatment; B. Cluster heat map of differential metabolites between Nipponbare and H570 after Al treatment.
